# Evolution of bidirectional sex change and gonochorism in fishes of the gobiid genera *Trimma*, *Priolepis*, and *Trimmatom*

**DOI:** 10.1007/s00114-017-1434-z

**Published:** 2017-03-01

**Authors:** Tomoki Sunobe, Tetsuya Sado, Kiyoshi Hagiwara, Hisaya Manabe, Toshiyuki Suzuki, Yasuhisa Kobayashi, Makoto Sakurai, Shin-ichi Dewa, Midori Matsuoka, Akihiko Shinomiya, Kazuya Fukuda, Masaki Miya

**Affiliations:** 1 Laboratory of Fish Behavioral Ecology, Tateyama Station, Field Science Center, Tokyo University of Marine Science and Technology, 670 Banda, Tateyama, 294-0308 Japan; 2 Department of Zoology, Natural History Museum and Institute, 955-2 Aoba-cho, Chuo-ku, Chiba 260-8682 Japan; 3 Yokosuka City Museum, 95 Fukada-dai, Yokosuka, 238-0016 Japan; 4 Education Center, Kagoshima University, Korimoto, Kagoshima, 890-0065 Japan; 5 Kawanishi-Midoridai Senior High School, Koyodai, Kawanishi, Hyogo 666-0115 Japan; 6 Department of Fisheries, Faculty of Agriculture, Kindai University, Nara, 631-8505 Japan; 7 Kagoshima Immaculate Heart College, 4-22-1 Toso, Kagoshima, 890-8525 Japan; 8 Diving Service Umi-Annai, 7-7 Masagohonmachi, Kagoshima, 890-0067 Japan; 9 Faculty of Fisheries, Kagoshima University, Kagoshima, 890-0056 Japan

**Keywords:** Bidirectional sex change, Phylogenetic analysis, Size-advantage model, Low-density model, Mating system, Gobiidae

## Abstract

Size-advantage and low-density models have been used to explain how mating systems favor hermaphroditism or gonochorism. However, these models do not indicate historical transitions in sexuality. Here, we investigate the evolution of bidirectional sex change and gonochorism by phylogenetic analysis using the mitochondrial gene of the gobiids *Trimma* (31 species), *Priolepis* (eight species), and *Trimmatom* (two species). *Trimma* and *Priolepis* formed a clade within the sister group *Trimmatom*. Gonadal histology and rearing experiments revealed that *Trimma marinae*, *Trimma nasa*, and *Trimmatom* spp. were gonochoric, whereas all other *Trimma* and *Priolepis* spp. were bidirectional sex changers or inferred ones. A maximum-likelihood reconstruction analysis demonstrated that the common ancestor of the three genera was gonochoristic. Bidirectional sex change probably evolved from gonochorism in a common ancestor of *Trimma* and *Priolepis.* As the gonads of bidirectional sex changers simultaneously contain mature ovarian and immature testicular components or vice versa, individuals are always potentially capable of functioning as females or males, respectively. Monogamy under low-density conditions may have been the ecological condition for the evolution of bidirectional sex change in a common ancestor. As *T. marinae* and *T. nasa* are a monophyletic group, gonochorism should have evolved from bidirectional sex change in a common ancestor.

**Electronic supplementary material:**

The online version of this article (doi:10.1007/s00114-017-1434-z) contains supplementary material, which is available to authorized users.

## Introduction

The evolution of sequential hermaphroditism in fish has been studied in relation to a central hypothesis called the size-advantage (SA) model. This model predicts that protogyny (sex change from female to male) and protandry (sex change from male to female) are favored by polygynous and random mating, respectively (Ghiselin 1969; Warner 1975, 1984). The reproductive success of larger males may be higher than that of smaller males in a polygynous system, resulting from female mate choice for larger males or male–male competition. Protogynous sex change is adaptive under this condition, with fish functioning as females at a smaller size and as males at a larger size. In contrast, male reproductive success is equal among size classes under conditions in which both sexes mate randomly with no mate choice. Protandrous sex change is favored under this condition because female fecundity increases linearly with size, and reproductive success is usually higher in larger females than in males of the same size. Empirical studies (e.g., in Labridae, Platycephalidae, Pomacanthidae, Pomacentridae, and Serranidae) show that these predictions correspond well with the observations (Kuwamura and Nakashima 1998; Munday et al. 2006).

In addition to these types of sex change, information on bidirectional sex change (sex change from female to male and male to female) has been accumulating in Epinephelinae (Serranidae), Cirrhitidae, Gobiidae, Labridae, Pomacanthidae, Pomacentridae, and Pseudochromidae (Munday et al. 2010; Kuwamura et al. 2015). *Labroides dimidiatus* (Labridae)*, Centropyge ferrugata* (Pomacanthidae), *Dascyllus aruanus* (Pomacentridae), and *Cirrhitichthys falco* (Cirrhitidae) are harem polygynous species that change sex from female to male when a dominant male disappears, as in protogynous fishes. However, sex change from male to female takes place in the smaller male of male–male pair under low-density conditions induced by experimental removing a female (Kuwamura et al. 2011, 2014, 2015) or natural disappearance of females (Kadota et al. 2012). In the monogamous coral dwelling gobiid fish *Paragobiodon echinocephalus* and *Gobiodon histrio*, bidirectional sex change takes place in male–male and female–female pairs. This system is adaptive if the closest individual is of the same sex after loss of a mate because long-distance movement between host corals may risk increased predation (Kuwamura et al. 1994; Munday 2002).

These studies explain how these types of sex change are adaptive in each social system. However, such observations do not explain the genealogical aspects of hermaphroditism. Sequential and simultaneous hermaphrodites have been reported in 27 teleost families of seven orders (Sadovy de Mitcheson and Liu 2008). Large-scale fish phylogeny studies indicate that hermaphroditism evolved independently in various taxa (Ross 1990; Mank et al. 2006). Fortunately, recent advances in phylogenetic analysis have clarified historical aspects of hermaphroditism in detail (Erisman et al. 2013). Protogynous sex change in the humbug damselfish (*Dascyllus*) may have evolved once in the ancestor of the genus, and the ability to change sex was lost in the ancestor of one of the clades (MacCafferty et al. 2002). Reconstruction of the phylogeny of the sexual patterns in Serranidae showed that gonochorism and simultaneous hermaphroditism evolved from protogyny as the ancestral condition (Erisman et al. 2009; Erisman and Hastings 2011). A comparative phylogenetic tree analysis in Epinephelinae (Serranidae) and Labridae indicated that the type of mating system, either polygyny or group spawning, is important in the evolution of protogyny or gonochorism, respectively, as predicted by the SA model (Molloy et al. 2007; Erisman et al. 2009; Kanzancioğlu and Alonzo 2010).

*Trimma*, *Priolepis*, and *Trimmatom* are small colorful gobiid fishes, including 92, 34, and seven valid species, respectively, distributed on rocky and coral reefs of temperate and tropical waters in the Indo-Pacific Ocean; *Priolepis* also has an extended distribution into the Atlantic Ocean (Winterbottom and Emery 1981; Winterbottom 2001; Nogawa and Endo 2007; Hoese and Larson 2010; Suzuki et al. 2012; Winterbottom et al. 2015). Winterbottom et al. (2014) analyzed cryptic *Trimma* spp. using partial nucleotide sequences from the cytochrome *c* oxidase I (COI) gene and revealed 94 potential species. These genera are very closely related and comprise a monophyletic group in Gobiidae (Winterbottom and Burridge 1992; Thacker 2009).

Bidirectional sex change has been reported previously in *T. grammistes*, *T. kudoi*, *T. okinawae*, *T. yanagitai*, *P. akihitoi*, *P. cincta*, *P. latifascima*, and *P. semidoliata*. In most cases, larger individuals of these species change to males and smaller individuals change to females in female–female and male–male pairs, respectively (Sunobe and Nakazono 1993; Shiobara 2000; Manabe et al. 2008, 2013; Sakurai et al. 2009). Harem polygyny is the *Trimma* *okinawae* mating system under natural conditions. Sex change from female to male occurs after the male disappears or becomes solitary; a solitary male changes sex to female when it joins another group as a subordinate (Sunobe and Nakazono 1990; Manabe et al. 2007). The *P. cincta* mating system is monogamous (Sunobe and Nakazono 1999), and male–female pairs of *P. akihitoi* and *P. semidoliata* appear in caves or rocky crevices, suggesting a monogamous system (Manabe et al. 2013). The gonads of these species simultaneously comprise ovarian and testicular portions, which are apparently separated by a thin wall of connective tissue (Sunobe and Nakazono 1993; Shiobara 2000; Manabe et al. 2008, 2013; Sakurai et al. 2009). Our recent study revealed that *Trimma* *marinae* is a gonochore with a monogamous mating system (Fukuda et al. 2017). However, no information is available on the sexuality or reproductive ecology of *Trimmatom*.

In this study, we analyzed the phylogenetic relationships of *Trimma* (31 species), *Priolepis* (eight species), and *Trimmatom* (two species), based on the nucleotide sequences from the mitochondrial ND4/5 gene region. We also present the sexuality of these species based on gonadal histology and rearing experiments. Lastly, we discuss the historical transitions of sexuality and the mating systems of species in these three genera.

## Materials and methods

### Taxon sampling

To reconstruct the evolution of bidirectional sex change and gonochorism in the three closely related gobiid genera, we sampled 31, eight, and two species of *Trimma*, *Priolepis*, and *Trimmatom*, respectively. We also sampled additional seven species as gobioid outgroups, and final rooting of the tree was made with a member of one of the most basally diverged families (Rhyacichthyidae: *Rhyacichthys aspro*) (Thacker 2009; Aggorreta et al. 2013) (Supplementary Table 1).

### DNA methods

The mitochondrial ND4/ND5 gene region outperforms commonly used mtDNA genes, such as COI, cyt *b*, and 12S/16S rRNA genes in phylogenetic analyses at broad taxonomic scales because it is relatively long (ca. 3.4 kb) and contains more phylogenetically informative variation at the first and second codon positions (Miya and Nishida 2000; Miya et al. 2006). Accordingly, we designed new polymerase chain reaction (PCR) primers to effectively amplify the gobioid ND4/ND5 region with reference to available whole gobioid mitogenomic sequences (Miya et al. 2003, 2013; Supplementary Fig. 1). We generated new ND4/5 gene region sequences from the 48 species using a combination of long and short PCR and direct sequencing techniques following the protocol suggested by Miya et al. (2006) (Supplementary Table 2).

### Phylogenetic analysis

Nucleotide sequences from the 48 species were concatenated and subjected together to multiple alignment using MAFFT ver. 6 (Katoh and Toh 2008). Unambiguously aligned sequences (total, 3679 bp) were used to construct a dataset that excluded quickly saturated transitional changes in the third codon position by converting purine (A/G) and pyrimidine (C/T) nucleotides to A and C, respectively, following Saitoh et al. (2006). Only transversions are considered by retaining all available positions in the dataset, so “noise” is effectively removed and the apparent loss of signals is avoided. The dataset was divided into four partitions (three partitions for the protein-coding genes and one partition for the tRNA genes) and subjected to partitioned maximum-likelihood (ML) analysis using RAxML ver. 7.2.8 (Stamatakis 2006). A general time-reversible model with sites following a discrete gamma distribution (GTR + *Γ*) was used, and a rapid bootstrap analysis was conducted with 1000 replications (–f a option).

### Tracing character evolution

The evolution of bidirectional sex change and gonochorism was reconstructed based on the best-scoring ML tree under the ML optimality criterion using Mesquite ver. 2.6 (Maddison and Maddison 2010). Two character states of sexuality were assigned: gonochorism (character state 0) and confirmed or likely bidirectional sex change (state 1).

### Determining sexuality

**Fig. 1:**
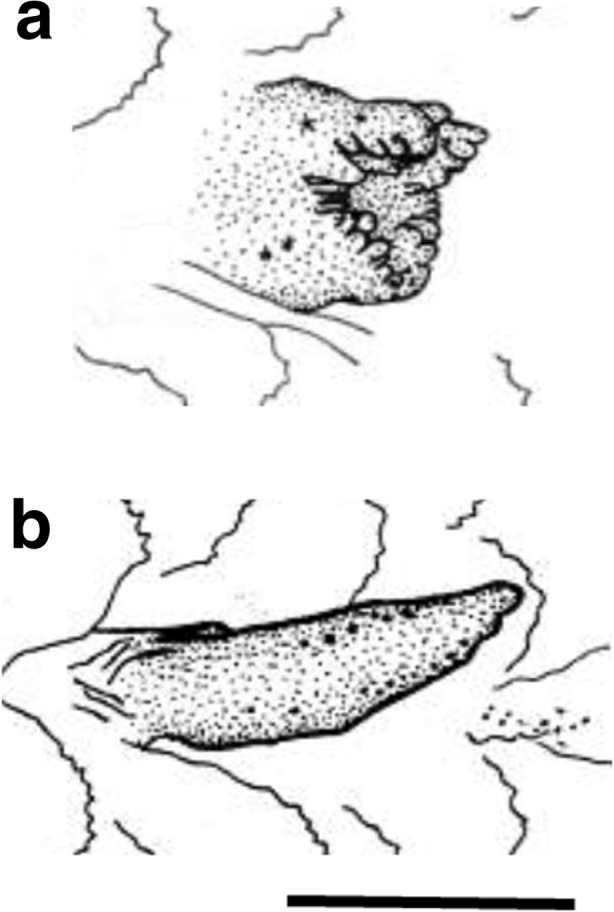
Urogenital papillae of female (**a**) and male (**b**) in *Trimma kudoi*. *Scale* 1 mm

Rearing experiments were conducted with *T. caesiura*, *T. maiandros*, *T. naudei*, and *Trimmatom* sp. to confirm sex change. Five, three, and three *T. caesiura*, *T. naudei,* and *T. maiandros* individuals, respectively, were collected on April 14 and 15, 2014, at Atetsu, Amami Island, Japan, and 12 *Trimmatom* sp. were collected on May 8 and 9, 2009, at Tsuchihama, Amami Island, Japan. The fish were brought to the laboratory, anesthetized in 100 ppm quinaldine, measured in total length (TL) to the nearest 0.1 mm, and identified by body size. Sex was determined from the shape of urogenital papilla: bulbous with several processes at the opening in females or tapered posteriorly in males, as determined in other gobiid species (Sunobe and Nakazono 1993; Kuwamura et al. 1994; Shiobara 2000; Manabe et al. 2008, 2013; Sakurai et al. 2009) (Fig. 1 shows urogenital papillae of *T. kudoi*, which is conspecific with *Trimma* sp. in Manabe et al. [2008]).

*T. caesiura*, *T. maiandros*, and *T. naudei* specimens were held together in their respective aquaria (60 × 35 × 30 cm) (Table 1). The sexual functions of the individuals were determined by spawning behavior, and the males were removed and placed in another aquarium (60 × 35 × 30 cm). If sex change to male was confirmed among the remaining females, the male previously removed was returned to its former aquarium. Subsequent observations revealed whether male-to-female sex change had occurred.

**Table 1:** Results of the *Trimma caesiura*, *Trimma maiandros*, and *Trimma naudei* rearing experiments

Individual name (mm TL)	Date of spawning	Sex role	Date of removal	Date of spawning	Sex role	Date of return	Date of spawning	Sex role	TL at end
*Trimma caesiura*									
TC1 (31.0)		Male	May 18			June 7		Male	35.0
TC2 (26.5)	May 9	Female			Male		June 28	Female	33.5
TC3 (23.0)	May 14	Female		May 24	Female		June 10	Female	27.6
*Trimma maiandros*									
TM1 (27.0)		Male	May 16			May 23		Male	29.0
TM2 (26.0)	May 16	Female			Male		May 31	Female	27.5
TM3 (23.0)	April 29	Female		May 19	Female		May 23	Female	25.5
*Trimma naudei*									
TN1 (34.0)		Male	May 13			June 7	June 26	Female	35.5
TN2 (33.5)	May 7	Female			Male			Male	37.0
TN3 (32.0)	May 2	Female		May 16	Female		June 8	Female	34.0
TN4 (28.0)	May 1	Female		May 20	Female		June 8	Female	31.0

Six male and six female *Trimmatom* sp. were kept in male–female pairs in six aquaria (30 × 20 × 23 cm) (Table 2). They were reared until spawning to confirm sexual function. The fish were then exchanged among the pairs to establish male–male and female–female pairs for 1 month. We observed whether spawning occurred or not by sex change. If spawning was not observed, sex was determined by the structure of the urogenital papilla and gonadal histology.

**Table 2:** Results of the *Trimmatom* sp. rearing experiments

Individual name (mm TL)	Shape of urogenital papilla at start	Date of spawning	Sex role	Exchange	Individual name	Shape of urogenital papilla at end	Gonad histology
Tom1 (23.0)	Tapered	June 30	Male	Pairing with the same sex	Tom1	Tapered	Testis
Tom2 (22.0)	Bulbous		Female		Tom3	Tapered	Testis
Tom3 (23.0)	Tapered	July 30	Male		Tom2	Bulbous	Ovary
Tom4 (20.5)	Bulbous		Female		Tom4	Bulbous	Ovary
Tom5 (28.0)	Tapered	May 30	Male	Pairing with the same sex	Tom5	Tapered	Testis
Tom6 (28.0)	Bulbous		Female		Tom7	Tapered	Testis
Tom7 (25.0)	Tapered	June 2	Male		Tom6	Bulbous	Ovary
Tom8 (24.0)	Bulbous		Female		Tom8	Bulbous	Ovary
Tom9 (24.0)	Tapered	July 21	Male	Pairing with the same sex	Tom9	Tapered	Testis
Tom10 (24.0)	Bulbous		Female		Tom11	Tapered	Testis
Tom11 (22.0)	Tapered	July 25	Male		Tom10	Bulbous	Ovary
Tom12 (23.0)	Bulbous		Female		Tom12	Bulbous	Ovary

Water in all aquaria was circulated continuously through gravel filters and maintained at 24–28 °C. Fish were fed formula food and *Artemia salina* larvae. A half-cut vinyl chloride pipe (5 cm inner diameter and 5 cm length) was added to each aquarium as a spawning nest.

We examined the gonads of the species listed, except those of *T. grammistes*, *T*. *kudoi*, *T. okinawae*, *T*. *yanagitai,* and eight *Priolepis* spp., whose gonadal structures have been published (Sunobe and Nakazono 1993; Shiobara 2000; Manabe et al. 2008, 2013; Sakurai et al. 2009; Cole 2010; Supplementary Table 1). Specimens of *T. caudomaculatum*, *T. flavatram, T. hayashii*, *T. maiandros*, *T. marinae*, *T. milta*, *T. taylori*, *Trimmatom* sp., *Trimmatom pharus*, *Cabillus* sp., and *Bathygobius fuscus* were collected by hand net using scuba or by snorkeling, and *Trimma rubromaculatum* was obtained from an ornamental fish shop. The fish were brought to the laboratory, anesthetized in 100 ppm MS-222, measured in TL to the nearest 0.5 mm, and sexed by the above methods. These specimens were fixed in Bouin’s solution for 24 h and preserved in 70% ethanol. The abdomens were embedded in paraffin, and whole gonads were sectioned serially at 5 μm and stained with hematoxylin and eosin. We examined the gonads of 19 *Trimma* spp. and *T. pharus* specimens deposited in the Royal Ontario Museum, the Yokosuka City Museum, and Kanagawa Prefectural Museum of Natural History (Supplementary Table 1). We dissected and extracted the abdominal organs containing the gonads after determining sex by the urogenital papilla structure and prepared the tissues following the method outlined above.

## Results

### Phylogenetic relationships

**Fig. 2:**
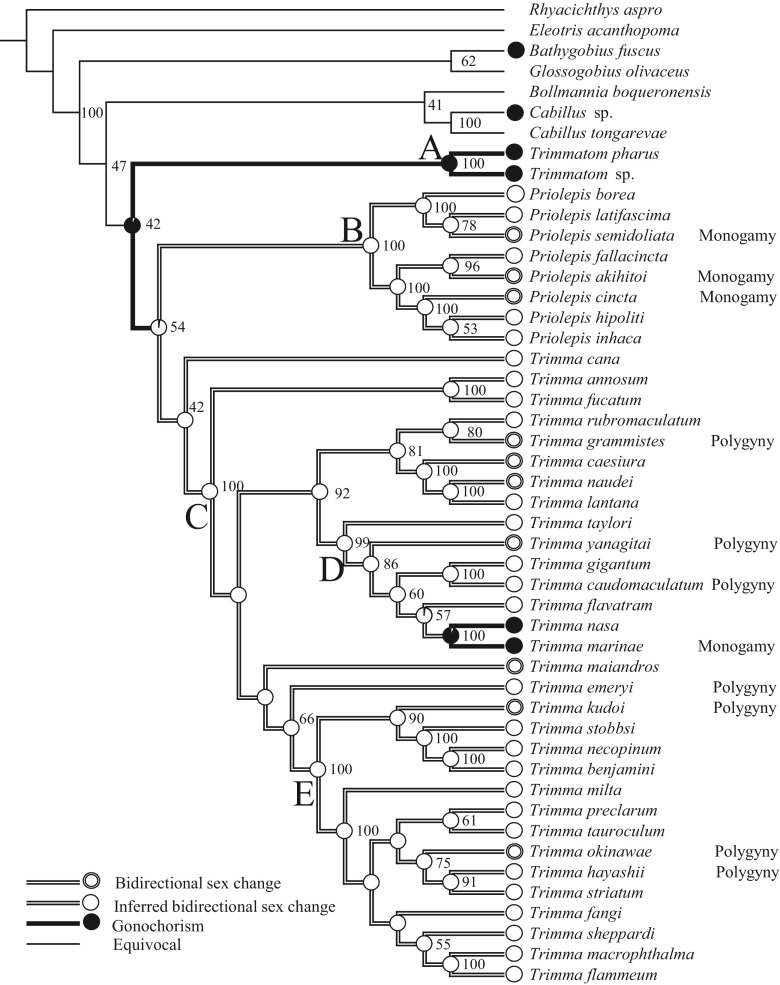
Phylogenetic tree for the maximum likelihood analysis and reconstruction of the evolution of bidirectional sex change and gonochorism. *Numbers* next to the *branches* indicate bootstrap probabilities ≥40% based on 1000 replications

*Trimma*, *Priolepis*, and *Trimmatom* were recovered together as a monophyletic group in the ML tree by only 42% bootstrap probability (BP), and *Trimma* and *Priolepis* formed a clade within the sister group *Trimmatom* with a 54% BP value. However, monophyly of each genus was strongly supported by a 100% BP value (clades A, B, and C) when the most basally diverged *Trimma* *cana* was excluded from the genus (Fig. 2).

### Rearing experiments and gonad histology

**Fig. 3:**
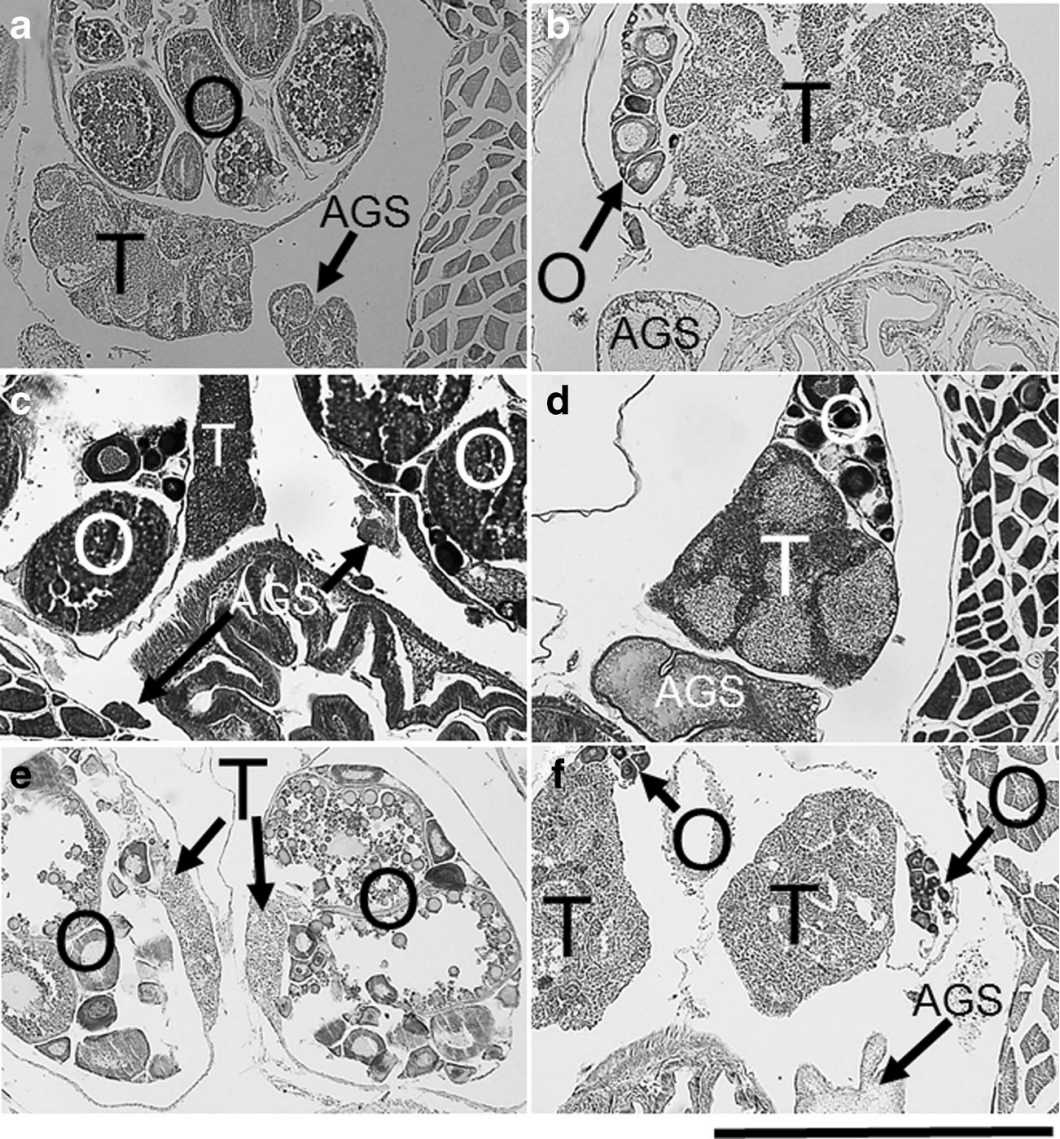
Gonadal structures of females (**a**, **c**, **e**) and males (**b**, **d**, **f**) in *Trimma caesiura* (**a**, **b**), *Trimma hayashii* (**c**, **d**), and *Trimma sheppardi* (**e**, **f**). *O* ovary, *T* testis, *AGS* accessory gonadal structure. *Scale* 0.5 mm

The *T. caesiura*, *T. maiandros,* and *T. naudei* rearing experiments showed that larger and smaller individuals functioned as males and females, respectively. After removing the males (TC1, TM1, and TN1), the largest females (TC2, TM2, and TN2) changed sex to males. When the males were returned, TC2, TM2, and TN1 changed back to females. These observations indicate that these species change sex bidirectionally and that their sex role is determined by body size; larger fish are males and smaller fish are females (Table 1). The gonadal structures in these species simultaneously consisted of both ovarian and testicular portions separated by a thin wall of connective tissue and an accessory gonadal structure (AGS; Cole 1990, 2010) (Fig. 3a, b shows *T. caesiura* gonads as an example). In female, the ovary contained oocytes in various stages of development. The testis and AGS were undeveloped (Fig. 3a). In male, the testis was filled with spermatozoa. The AGS was developed, and ovarian tissue was filled with young oocytes (Fig. 3b). The same gonadal structure as that described for the above species was also found in the other *Trimma* spp., except *T. marinae* and *T. nasa* (Fig. 3c–f shows *T. hayashii* and *T. sheppardi* gonads as examples).

**Fig. 4:**
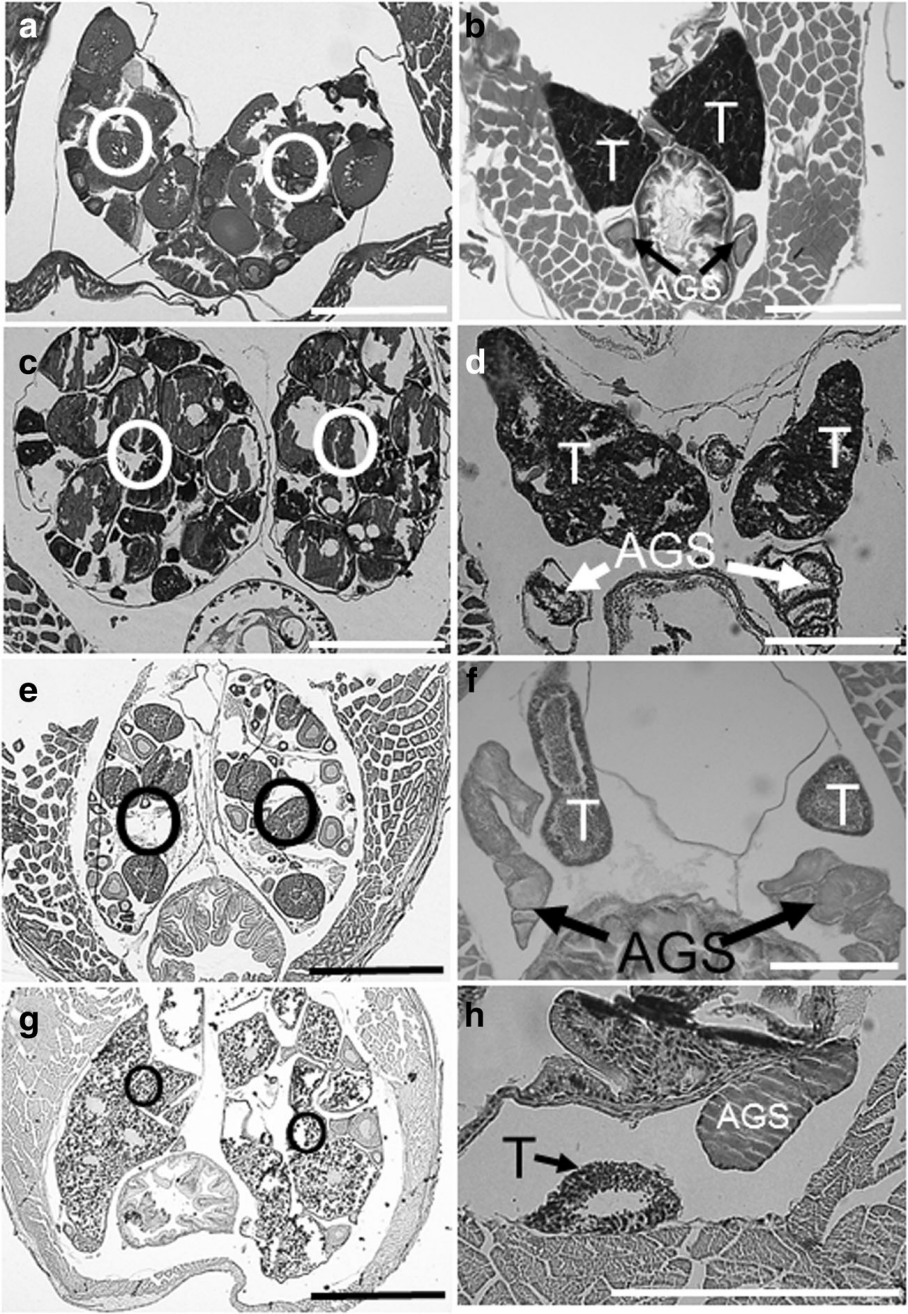
Gonadal structures of females (**a**, **c**, **e**, **g**) and males (**b**, **d**, **f**, **h**) in *Trimma marinae* (**a**, **b**), *Trimma nasa* (**c**, **d**), *Trimmatom* sp. (**e**, **f**) and *Trimmatom pharus* (**g**, **h**). *O* ovary, *T* testis, *AGS* accessory gonadal structure. *Scale* 0.5 mm (**a**, **b**, **c**, **d**, **e**, **g**) and 0.2 mm (**f**, **h**)

No spawning was observed in any pair during the *Trimmatom* sp. exchange experiments. At the end, shape of urogenital papillae of the individuals did not change, and there was no signal for sex change by gonad histology (Table 2). We did not detect intersexual gonads in *T. marinae*, *T. nasa, Trimmatom* sp., and *T. pharus*. The male gonads consisted of testes with the AGS, and the female gonads are composed of only ovaries (Fig. 4). The gonadal structures of outgroup species *Cabillus* sp. and *B. fuscus* were the same as those of these four species.

## Discussion

### Phylogenetic relationships

Thacker (2009) analyzed the phylogeny of Gobioidei using the ND1/2 and COI regions and reported that *Trimma caesiura*, *Priolepis cincta, Priolepis* *eugenius,* and *Trimmatom eviotops* form a monophyletic group with strong support. These results suggest a close relationship among these three genera. Winterbottom et al. (2014) presented the relationships among cryptic *Trimma* species using partial COI gene nucleotide sequences. Species in clade D, except *T. gigantum* and *T. caudomaculatum*, and those in clade E (Fig. 2), which were supported by a high BP value, corresponded with the grouping reported by Winterbottom et al. (2014), although the detailed topologies within the group differed. The concordance of these results using different methods suggests that the relationships among these species have been correctly deduced.

As mentioned above, there are 92, 34, and seven valid species in *Trimma*, *Priolepis*, and *Trimmatom*, respectively, but in this study, we examined only 31, eight, and two species, respectively. In addition, we did not investigate the related genera *Egglestonichthys* and *Paratrimma* (Winterbottom and Burridge 1992) due to difficulty to obtain specimens. Future studies will examine their phylogenetic relationships by analyzing a greater number of species and genera, using other genes as well as mitochondrial gene ND4/5.

### Determination of sexuality

The results show that *T. caesiura*, *T. maiandros,* and *T. naudei* exhibited bidirectional sex change, and the gonadal structures simultaneously consisted of both ovarian and testicular portions. The same gonadal structures are also reported in *T. grammistes*, *T*. *kudoi*, *T. okinawae*, *T*. *yanagitai*, *P. akihitoi*, *P. cincta*, *P. latifascima,* and *P. semidoliata* in which bidirectional sex change has been confirmed previously (Sunobe and Nakazono 1993; Shiobara 2000; Manabe et al. 2008, 2013; Sakurai et al. 2009).

This type of gonadal structure is also found in the male phase of protandrous species, *Amphiprion akallopisos* (Pomacentridae), *Acanthopagrus schlegelii* (Sparidae), and *Thysanophrys celebica* (Platycephalidae). The testicular part is active, while the ovarian part is inactive. However, the testicular part disappears after sex change from male to female, and the active ovarian part remains (Fricke and Fricke 1977; Chang and Yueh 1990; Sunobe et al. 2016).

A similar gonadal structure is reported in the simultaneous hermaphrodites *Serranus scriba* (Serranidae) and *Kryptolebias marmoratus* (Rivulidae). Gonads of these species are composed of simultaneously active ovarian and testicular part. In these species, one individual can function as male and female at the same time (Tuset et al. 2005; Sakakura et al. 2006).

Although both ovary and testis are present at the same time in one individual in these *Trimma* and *Priolepis* spp., either the ovary or the testis is active or inactive in male and female phases, respectively (Fig. 3), unlike the above protogynous and simultaneous hermaphrodite species. As the ovarian part remains after sex change from female to male, the male can revert to female.

The same gonadal structure is also found in the other *Trimma* spp., except *T. marinae* and *T. nasa* in this study, and in *P. borea*, *P. fallacincta*, *P. hipoliti*, and *P. inhaca* (Cole 2010; Manabe et al. 2013). Although we did not confirm bidirectional sex change in field observations or rearing experiments on these species, they are inferred to exhibit bidirectional sex change.

Cole (2010) shows that a precursor AGS (pAGS) is detectable in the ovary of protogynous gobiid fish (*Bryaninops*, *Elacatinus*, *Fusigobius,* and *Lophogobius*) as a valid indicator of protogyny. In *T. marinae*, *T. nasa,* and *Trimmatom* sp., intersexual gonads were not detected, and the female gonads are composed of only ovaries without a pAGS (Fig. 4a–f). *T. marinae* did not change sex in male-only or female-only groups after a 2-month rearing experiment (Fukuda et al. 2017). Female *T. nasa* are larger than males because growth rate of the former is faster than that of the latter (Winterbottom and Southcott 2008), not showing protandrous sex change. At the end of *Trimmatom* sp. exchange experiments, any individual did not spawn, and there was no evidence suggesting sex change from either changes in the papillae or gonad histology (Table 2). These data strongly suggest that three species are gonochores. Although we did not conduct *T. pharus* rearing experiments, we regard this species as a gonochore because the gonadal structures of all specimens examined were the same as those of the above species (Fig. 4g, h). For sexuality of the outgroup species, *Cabillus* sp. and *B. fuscus* should be identified as a gonochore by lack of pAGS in female gonads.

The conclusion for determination of sexuality on the above species is listed in Fig. 2.

### Evolution of bidirectional sex change and gonochorism and the ancestral mating system

Here, we show the nodes at which bidirectional sex change or gonochorism evolved, although the proposed phylogenetic hypothesis potentially could be improved, as mentioned above.

As a common ancestor of *Trimma*, *Priolepis*, and *Trimmatom* was predicted as a gonochore, the evolution of bidirectional sex change from gonochorism should occur in a *Trimma* and *Priolepis* common ancestor (Fig. 2). Although there is no information on sexuality of outgroup species other than *Cabillus* sp. and *B. fuscus*, these data suggest that the common ancestors of *Trimma*, *Priolepis*, *Trimmatom* and these outgroup species are gonochores. The theoretical and empirical studies indicate that mating system is one of the main selection pressures favoring sex change (Ghiselin 1969; Warner 1975, 1984; Kuwamura and Nakashima 1998; Munday et al. 2006, 2010; Sadovy de Mitcheson and Liu 2008). We address the ecological condition for evolution of bidirectional sex change in the following cases to discuss which type of mating system was adopted by the common ancestor.

In polygynous species exhibiting bidirectional sex change, such as *L. dimidiatus*, *D. aruanus*, *C. ferrugata*, and *C. falco*, females change to males after the dominant male disappears or if a branching harem forms (Kuwamura 1981; Coates 1982; Sakai 1997; Kadota et al. 2012). Female mate choice for larger males or male–male competition, which favors protogyny as predicted by the SA model, should occur in populations which individuals frequently interact. In this condition, a male can monopolize several females, and males compete with resources or females. Male-to-female sex change occurs by establishing a pair between the nearest or second-nearest males after experimental removal or natural disappearance of females. The reverse sex change condition seems to correspond with low density (Kuwamura et al. 2011, 2014, 2015; Kadota et al. 2012). As territorial males rarely lose their mates, such a low-density condition could occur near the edge of the distribution (Kuwamura et al. 2011). These results suggest that female-to-male sex change evolved first in the center of the distribution and that male-to-female sex change was favored later.

Monogamous gobiid fish, such as *P. echinocephalus* and *G. histrio*, are obligate coral-dwelling species. These species exhibit female-to-male and male-to-female sex change by mating with individuals of the same sex. If the mate is lost or the coral dies, it is more advantageous for both sexes to re-establish the pair with a nearby consensual individual than to search for a heterosexual fish over a long distance because it may increase predation risk (Kuwamura et al. 1994; Nakashima et al. 1995; Munday 2002). Ghiselin (1969) proposed a “low-density model” in which simultaneous hermaphroditism is adaptive in a species with low mobility or low population density to reduce mating opportunities. The above cases are very consistent with this model. As any individual could function as a male or a female, the evolution of female-to-male and male-to-female (bidirectional) sex change may have occurred simultaneously.

*T. okinawae*, *T. grammistes*, *T. kudoi*, *T. yanagitai*, *T. caudomaculatum*, *T. hayashii*, and *T. emeryi* inhabit groups of more than three individuals, and the mating system is polygynous (Sunobe and Nakazono 1990; Shiobara 2000; Manabe et al. 2007, 2008; Sakurai et al. 2009; Sunobe unpublished data; see also Fig. 2). Meanwhile, *P. akihitoi*, *P. cincta,* and *P. semidoliata* appear as a pair or singly and are monogamous under a low-density condition (Sunobe and Nakazono 1999; Manabe et al. 2013; see also Fig. 2).

Although data on the mating system for both genera are limited, that of the common ancestor would have been either polygyny or monogamy. Figure 2 indicates that bidirectional sex change evolved from gonochorism without protogyny. The structure of intersexual gonads (Fig. 3) indicates that an individual could function as a male or a female under any condition. Therefore, the ecological conditions for evolution of bidirectional sex change in the common ancestor may correspond to the latter case above, that is, monogamy under low-density conditions.

*T. okinawae* is harem polygynous and is the only species in which bidirectional sex change was confirmed under natural conditions among *Trimma* spp. Female-to-male sex change occurs when the dominant male disappears or the female loses its mate. Male-to-female sex change is observed in bachelor males after mating with larger males (Manabe et al. 2007). The conditions for sex change are primarily the same as those in other polygynous species, as mentioned above. However, Fig. 2 shows that bidirectional sex change was not independently favored in *T. okinawae* compared to other related species. Polygyny may have evolved during speciation from the common ancestor to *Trimma*, but the trait for male-to-female sex change was not lost. It may be adaptive for *T. okinawae* to retain the ability to change sex bidirectionally because the study described above indicates that a low-density condition occurs occasionally.

Figure 2 shows that gonochorism should have evolved from bidirectional sex change in the common ancestor of the gonochoristic species *T. marinae* and *T. nasa*. Evolution from hermaphroditism to gonochorism has been reported in *Dascyllus* (Pomacentridae), Labridae, Serranidae, and Sparidae (McCafferty et al. 2002; Erisman et al. 2009, 2013; Erisman and Hastings 2011). A pairing *T. marinae* female occupies a male and excludes other females, resulting in monogamy, because the ability to care paternally may be limited to one clutch. There are two explanations for the adaptive significance of gonochorism in this species. First, as this species forms aggregations, both sexes can easily mate with a new mate without changing sex. Second, it may be costly to change sex due to a short lifespan because an individual cannot spawn during the sex change period (Fukuda et al. 2017). Data on the reproductive ecology of the species in clade D (Fig. 2) are needed to clarify the evolution of gonochorism in the common ancestor of *T. marinae* and *T. nasa*. However, data on *T. taylori*, *T. gigantum*, *T. flavatram,* and *T. nasa* are unavailable. Thus, further study is needed to clarify the reproductive ecology of these species.

## Electronic supplementary material

ESM 1 (PDF 174 kb)
