# Supplementary material for: Evolution of bidirectional sex change and gonochorism in fishes of the gobiid genera Trimma, Priolepis, and Trimmatom
Source: Naturwissenschaften. 2017 Mar 1;104(3):15. doi: 10.1007/s00114-017-1434-z (PMC5332495; doi:10.1007/s00114-017-1434-z)
Supplement: Supplementary file 1 — (PDF 174 kb) [file 114_2017_1434_MOESM1_ESM.pdf]

Supplementary file

Journal: The Science of Nature

Evolution of bidirectional sex change and gonochorism in fishes of the gobiid genera *Trimma*, *Priolepis*, and *Trimmatom*

Tomoki Sunobe[1*] ・Tetsuya Sado[2] ・Kiyoshi Hagiwara[3] ・Hisaya Manabe[4] ・Toshiyuki Suzuki[5] ・Yasuhisa Kobayashi[6] ・Makoto Sakurai[7] ・Shin-ichi Dewa[8] ・Midori Matsuoka[9] ・Akihiko Shinomiya[9] ・Kazuya Fukuda[1] ・Masaki Miya[2]

[1] Laboratory of Fish Behavioural Ecology, Tateyama Station, Field Science Center, Tokyo University of Marine Science and Technology, 670 Banda, Tateyama 294-0308, Japan;

[2] Department of Zoology, Natural History Museum and Institute, Chiba, 955-2 Aoba-cho, Chuo-ku, Chiba 260-8682, Japan;

[3] Yokosuka City Museum, 95 Fukada-dai, Yokosuka 238-0016, Japan;

[4] Education Center, Kagoshima University, Korimoto, Kagoshima 890-0065, Japan;

[5] Kawanishi-midoridai Senior High School, Kouyoudai, Kawanishi, Hyogo 666-0115, Japan;

[6] Department of Fisheries, Faculty of Agriculture, Kindai University, Nara 631-8505, Japan;

[7] Kagoshima Immaculate Heart College, 4-22-1 Toso, Kagoshima 890-8525, Japan;

[8] Diving Service Umi-Annai, 7-7 Masagohonmachi, Kagoshima 890-0067, Japan;

[9] Faculty of Fisheries, Kagoshima University, Kagoshima 890-0056, Japan.

*Corresponding author: sunobe@biscuit.ocn.ne.jp

Supplementary Table 1 A list of the species used in phylogenetic analysis and determination of sexuality. For non-catalogued specimens, locality or source is shown. CBM, Natural History Museum and Institute, Chiba; CMNH, Coastal Branch of Natural History Museum and Institute, Chiba; NMST, National Science Museum; ROM, Royal Ontario Museum; YCM, Yokosuka City Museum; KPM, Kanagawa Prefectural Museum of Natural History. *conspecific with *Trimmatom* sp. by Akihito et al. (2013)

Species	Accession number (Registration number)	Specimens for determination of sexuality (mm in TL : male; female)
Ingroup		
Trimma annosum	AB854445(CBM-ZF11489)	ROM45977 (23.0; 21.0)
T. benjamini	AB854447(ROM64354)	ROM4612 (25.0; 24.0)
T. caudomaculatum	AB854465	Amami Isl., Japan (32.0; 29.0)
T. caesiura	AB854419(CBM-ZF11460)	YCM-P34185 (36.0; 30.5)
T. cana	AB854449(ROM74764)	ROM74894 (24.0; 23.5)
T. emeryi	AB854421(CBM-ZF11462)	YCM-P36315 (22.0,22.5; 19.5, 20.5)
T. fangi	AB854453	ROM85152 (26.5; 26.0)
T. flammeum	AB854454	ROM59781 (26.0; 25.0)
T. flavatram	AB854432(CBM-ZF11475)	Amami Isl., Japan (female 25.0, 26.5)
T. fucatum	AB854455(ROM68769)	ROM58769 (25.5; 25.0)
T. gigantum	AB854456(ROM80658)	ROM88104 (33.0; 39.0)
T. grammistes	AB854422(CBM-ZF11463)	
T. hayashii	AB854431(CBM-ZF11474)	Amami Isl., Japan (24.0; 20.0)
T. kudoi	AB854434(CBM-ZF11477)	
T. lantana	AB854457	ROM83136 (22.0; 24.0)
T. macrophthalma	AB854424(CBM-ZF11465)	YCM-P37070 (20.0, 21.0; 19.0, 19.0)
T. maiandros	AB854430(CBM-ZF11473)	Amami Isl., Japan (22.0, 21.5)
T. marinae	AB854423(CBM-ZF11464)	Amami Isl., Japan (22.0; 25.0)
T. milta	AB854474(ROM80399)	Amami Isl., Japan (26.0; 23.0, 23.5)
T. nasa	AB854458(ROM80347)	ROM74780 (18.5; 21.5)
T. naudei	AB854425(CBM-ZF11466)	YCM-P41276 (33.5; 32.0, 35.0)
T. necopinum	AB854459	ROM52450 (26.5; 24.0)
T. okinawae	AB854426(CBM-ZF11467)	
T. preclarum	AB854460(ROM80021)	ROM 83062 (24.5; 24.0)
T. rubromaculatum	AB854436(CBM-ZF11479)	Ornament fish shop (22.0, 26.0; 19.0)
T. sheppardi	AB854427(CBM-ZF11468)	YCM-P28333 (24.0; 23.0)
T. stobbsi	AB854461(ROM63937)	ROM46052 (18.5; 20.0)
T. striatum	AB854462(ROM74959)	YCM-P37166 (26.0, 28.0; 24.5, 27.0)
T. tauroculum	AB854463(ROM80354)	ROM80027 (21.0; 21.5)
T. taylori	AB854428(CBM-ZF11469)	Amami Isl., Japan (27.0; 30.0)
T. yanagitai	AB854433(CBM-ZF11476)	
Priolepis akihitoi	AB854442(CBM-ZF11484)	
P. borea	LC021306/7 (CMNH-ZF016029)	
P. cincta	AB854437(CBM-ZF11480)	
P. fallacincta	AB854438(CBM-ZF11481)	
P. hipoliti	LC021302	
P. inhaca	AB854439(CBM-ZF11482)	
P. latifascima	AB854470(CBM-ZF12223)	
P. semidoliata	AB854440/1(CBM-ZF-11483)	
Trimmatom sp.*	AB854443(CBM-ZF11485)	Amami Isl., Japan (25.0); Kuchierabu Isl., Japan (23.0)
Trimmatom pharus	AB854471(CBM-ZF12297)	Iriomote Isl., Japan (17.0), YCM-P 44761 (17.0, 18.5), KPM-NI 5522 (15.5) ; Sesoko Isl., Japan (21.0), YCM-P 44761 (17.5, 19.5), KPM-NI 5071(16.0), 5076(17.0)
Gobiid outgroup		
Bathygobius fuscus	AB854444(CBM-ZF-11488)	Sakurajima, Kagoshima, Japan (female 78.0, 97.3)
Bollmannia boqueronsis	LC021303	
Cabillus tongarevae	AB854467 (NMST-P 75536)	
Cabillus sp.	AB854468 (CBM-ZF-12221)	Iriomote Isl., Japan (44.8; 44.0)
Gollogobius olivaceus	LC021304 (CBM-ZF 12215)	
Non-gobiid outgroup		
Eleotris acanthopoma	AP004455	
Rhyacichthys aspro	AP004454	

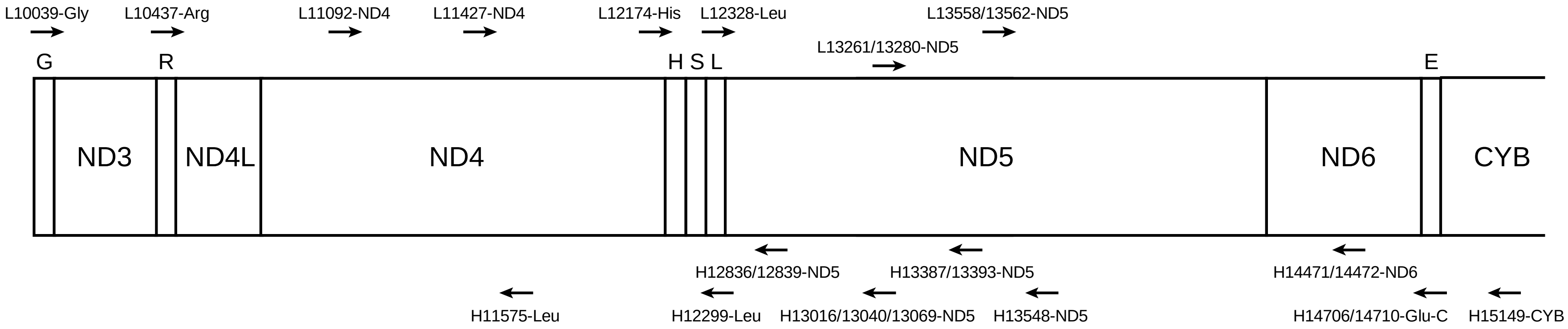

Supplementary Fig. 1 Schematic representation of the ND4/ND5 gene region and relative positions of the newly designed and previously published polymerase chain reaction primers (see Supplementary Table 2). Transfer RNA genes are represented by a single-letter code

Supplementary Table 2 Primers are designated by their 3´ ends, which correspond to the position on the human mitochondrial genome (Anderson et al. 1981) by convention. L and H denote heavy and light strands, respectively. Degenerate positions of sequences are denoted using IUPAC codes

Primer	Sequence (5´–3´)	Reference
Long PCR primers		
L10039-Gly-G	TTCCAATYWCMTGGTYTTGG	This study
L10437-Arg-G	TATTTTAATTAAAATAKTTGRTTTCG	This study
H14706-Glu-G	ACAACGRTGGTTTTTCAAG	This study
H14710-Glu-C	CTTGTAGTTGAATWACAACGGTGGTTYTTC	Miya et al. (2006)
H15149-CYB	GGTGGCKCCTCAGAAGGACATTTGKCCTCA	Miya and Nishida (2000)
Short PCR primers		
L10437-Arg-G	TATTTTAATTAAAATAKTTGRTTTCG	This study
L11092-ND4-G	TKTTCTATGTKATGTTTGARGC	This study
L11427-ND4-G	CCMAARGCSCATGTWGARGC	This study
L12174-His-G	TTTAAMKAAAACATTAGATTGTGATTC	This study
L12328-Leu-C	AACTCTTGGTGCAAMTCCAAG	Miya et al. (2006)
L12936-ND5	AACTCMTGGGAGATTCAACAA	Miya and Nishida (2000)
L13261-ND5-G	GCKTTTTCAACWTCAAGCCA	This study
L13280-ND5-M	CARCTWGGCCTAATRATRGT	This study
L13558-ND5-G	TCMTACCTMAACGCCTGAGC	This study
L13562-ND5	TCTTACCTAAACGCCTGAGCCCT	Miya and Nishida (1999)
H11575-ND4-G	ATKAGKGAYTTKAGGTCTGTTTG	This study
H12299-Leu-G	GCACCAAGAGTTTTTGGTTCC	This study
H12836-ND5-G	GCKGCKGTGTTKGCGTCKGC	This study
H12839-ND5-G	AGKGCTGCWGTGTTKGCRTC	This study
H13016-ND5	AGTCAKGGGTGGAGKCCRAATTG	Miya and Nishida (1999)
H13040-ND5-G	GGYGTWGGRCCCTCCATKGC	This study
H13069-ND5	GTGCTGGAGTGKAGTAGGGC	Miya and Nishida (1999)
H13387-ND5-G	TTKCGGATGTCYTGYTCRTC	This study
H13393-ND5-C	CCTATTTTKCGGATGTCTTGYTC	Miya et al. (2006)
H13548-ND5-G	GTKAGGGAKAGGGCTCAGGCGTT	This study
H14471-ND6	GCTTTGGCKGCKGAGCCTT	Minegishi et al. (2005)
H14472-ND6-G	GTKTTTGCTTATTCKKCRGC	This study
H14706-Glu-G	ACAACGRTGGTTTTTCAAG	This study
H14710-Glu-C	CTTGTAGTTGAATWACAACGGTGGTTYTTC	Miya et al. (2006)

References

Akihito, Sakamoto K, Ikeda Y, Aizawa, M. (2013) Gobioidei. In: Nakabo T (ed) Fishes of Japan with pictorial keys to the species third edition. Tokai University Press, Hadano-shi, Kanagawa, Japan, pp. 1347–1608

Anderson S, Bankier AT, Barrell BG, de Bruijn MHL, Coulson AR, Drouin J, Eperon IC, Nierlich DP, Roe BA, Sanger F, Schreier PH, Smith AJH, Staden R, Young IG (1981) Sequence and organization of the human mitochondrial genome. Nature 290:457–465

Minegishi Y, Aoyama J, Inoue JG, Miya M, Nishida M, Tsukamoto K (2005) Molecular phylogeny and evolution of the freshwater eels genus *Anguilla* based on the whole mitochondrial genome sequences. Mol Phylogenet Evol 34:134–146

Miya M, Nishida M (1999) Organization of the mitochondrial genome of a deep-sea fish, *Gonostoma gracile* (Teleostei: Stomiiformes): first example of transfer RNA gene rearrangements in bony fishes. Mar Biotechnol 1: 416–426

Miya M, Nishida M (2000) Use of mitogenomic information in teleostean molecular phylogenetics: a tree-based exploration under the maximum-parsimony optimality criterion. Mol Phylogenet Evol 17: 437–455

Miya M, Saitoh K, Wood R, Nishida M, Mayden RL (2006) New primers for amplifying and sequencing the mitochondrial ND4/ND5 gene region of the Cypriniformes (Actinopterygii: Ostariophysi). Ichthyol Res 53: 75–81
